# Effects of Chinese Gallotannins on Antioxidant Function, Intestinal Health, and Gut Flora in Broilers Challenged with *Escherichia coli* Lipopolysaccharide

**DOI:** 10.3390/ani14131915

**Published:** 2024-06-28

**Authors:** Yuemeng Fu, Peng Yuan, Nadia Everaert, Luke Comer, Shuzhen Jiang, Ning Jiao, Libo Huang, Xuejun Yuan, Weiren Yang, Yang Li

**Affiliations:** 1 Key Laboratory of Efficient Utilization of Non-Grain Feed Resources, Ministry of Agriculture and Rural Affairs, College of Animal Science and Technology, Shandong Agricultural University, Panhe Street 7, Tai’an 271017, China; fuyuemeng@yeah.net (Y.F.); yp8511@163.com (P.Y.); shuzhen305@163.com (S.J.); jiaoning@sdau.cn (N.J.); huanglibo@sdau.cn (L.H.); wryang@sdau.cn (W.Y.); 2 Division of Animal and Human Health Engineering, Department of Biosystems, KU Leuven, Kasteelpark Arenberg 30, 3001 Heverlee, Belgium; nadia.everaert@kuleuven.be (N.E.); luke.comer@kuleuven.be (L.C.); 3 College of Life Sciences, Shandong Agricultural University, Daizong Street 61, Tai’an 271018, China; xjyuan@sdau.cn

**Keywords:** broilers, intestinal inflammation, tannin, microorganisms, LPS

## Abstract

**Simple Summary:**

In intensive farms, broilers are easily infected by harmful bacteria, resulting in intestinal damage and affecting their health. The prohibition of antibiotics makes it necessary to find new antibacterial products, especially native substances. As a kind of traditional Chinese herbal medicine, Chinese gallotannins (CGT) containing tannins have antioxidant, anti-inflammatory, and bactericidal effects. Therefore, in this experiment, we established a model of intestinal injury in broilers by intraperitoneal administration of lipopolysaccharide (LPS) in *Escherichia coli* to explore the protective effect of CGT on intestinal injury in broilers induced by LPS challenge. The results show that CGT effectively alleviated intestinal mucosal injury and repaired the intestinal barrier effectively by repairing intestinal villus morphology, inhibiting apoptosis, decreasing pro-inflammatory factors, and stabilizing microbial ecology, thus raising the body weight to a normal level. A dietary supplementation of 300 mg/kg CGT might be a potential way to substitute antibiotics to attenuate intestinal injury induced by LPS in broilers.

**Abstract:**

This experiment was conducted to study the protective effects of dietary Chinese gallotannins (CGT) supplementation against *Escherichia coli* lipopolysaccharide (LPS)-induced intestinal injury in broilers. Four hundred and fifty healthy Arbor Acres broilers (one-day-old) were randomly divided into three groups: (1) basal diet (CON group), (2) basal diet with LPS challenge (LPS group), and (3) basal diet supplemented with 300 mg/kg CGT as well as LPS challenge (LPS+CGT group). The experiment lasted for 21 days. Intraperitoneal LPS injections were administered to broilers in the LPS group and the LPS+CGT group on days 17, 19, and 21 of the trial, whereas the CON group received an intraperitoneal injection of 0.9% physiological saline. Blood and intestinal mucosa samples were collected 3 h after the LPS challenge. The results showed that LPS administration induced intestinal inflammation and apoptosis and damaged small intestinal morphology and structure in broilers. However, dietary supplementation with CGT alleviated the deleterious effects on intestinal morphology and barrier integrity caused by the LPS challenge, while also reducing intestinal apoptosis and inflammation, enhancing intestinal antioxidant capacity, and increasing cecal microbial alpha diversity in the LPS-challenged broilers. Therefore, our findings demonstrated that a 300 mg/kg CGT addition could improve intestinal morphology and gut barrier structure, as well as maintaining bacterial homeostasis, in broilers exposed to LPS. This might partially be attributed to the reduced cell apoptosis, decreased inflammatory response, and enhanced antioxidant capacity in the small intestinal mucosa.

## 1. Introduction

Implementing intensive husbandry is essential to enhance the production efficiency of broilers, thereby yielding increased economic advantages [1,2]. As such, farming practices continue to be upscaled. However, intensive poultry farms also run the risk of both breeding and emitting potentially pathogenic microorganisms into the environment, thereby increasing the risk of poultry diseases in the wider population [3]. As the largest digestive and immune organ, the intestine is crucial for the absorption and transport of nutrients, meanwhile providing resistance against colonization by pathogens and harmful microorganisms [4,5]. As a result, pathogenic bacterial infection in the intestine can induce significant disruption, leading to intestinal damage and, ultimately, the decreased growth performance of broilers. With the prohibition of antimicrobial growth promoters and an increasing expectation for meat quality, there has been a significant emphasis on searching for natural and environmentally friendly additives to maintain gut health in broilers lately [6,7,8].

Tannins are polyphenolic compounds widely present in nature, which can further be classified into condensed tannins, hydrolyzable tannins (HT), and phlorotannins, according to their structures [9,10]. Chinese gallotannins (CGT) are a kind of HT produced by the Rhus chinensis Mill., popular in traditional Chinese medicine, and are present in the galls generated by aphid infestation of the leaves [11]. The application of CGT as a potential alternative to antibiotics in animal production has aroused widespread interest in recent years because of its antimicrobial, antioxidant, and anti-inflammatory activities. When compared to zinc oxide, CGT supplementation in the piglet diet has shown increased intestinal antioxidant capacity and resulted in the presence of fewer harmful gut bacteria [12]. Previous studies in broilers have demonstrated that dietary CGT supplementation could be beneficial to liver health status [13,14], improve intestinal development and function [15], and increase the abundance of beneficial bacteria [16]. Furthermore, tannic acid (TA) has been demonstrated to maintain the intestinal barrier integrity of broilers under *Salmonella pullorum*, *Clostridium perfringens*, and *Eimeria Maxima* challenge [17,18,19]. Additionally, it enhanced the immunity and antioxidant function of broilers suffering from necrotic enteritis [18]. Tannins extracted from *Acacia mearnsii*, *Castanea sativa*, *Schinopsis lorenzii*, and *Caesalpinia spinosa* function differently on antioxidants, immune function, and intestinal bacterial composition in broilers [20]. However, it remains unclear whether dietary supplementation of CGT can be effective in alleviating intestinal injury in broilers.

A high incidence of *Escherichia coli* (*E. coli*)-induced intestinal damage has been reported in modern poultry production [21,22]. The cell wall component lipopolysaccharide (LPS) is one of the main surface antigens of *E. coli* and is considered a main virulence factor [23]. Numerous studies have reported that an intraperitoneal injection of *E. coli* LPS may induce intestinal injury in broilers by promoting the formation of reactive oxygen species and pro-inflammatory cytokines [24,25,26].

Therefore, in the present study, an intestinal injury model via an *E. coli* LPS challenge was constructed to better understand the effects of dietary CGT supplementation on intestinal damage and to explore its potential value in broiler production.

## 2. Materials and Methods

### 2.1. Experimental Animals and Management

A total of 450 healthy and uniformly weighted (48.56 ± 0.10 g) 1-day-old Arbor Acres broilers (from the same batch) were divided at random into 3 treatment groups, namely the control (CON) group, LPS group, and LPS+CGT group with 6 replicates of 25 chickens per treatment. The broilers in the CON group and LPS group received a basal diet while those in the LPS+CGT group were provided with a basal diet with 300 mg/kg of supplemented CGT. The CGT product was microencapsulated and provided by Wufeng Chicheng Biotechnology Co., Ltd. (Yichang, China) with 40% effective concentrations. The optimum dosage of CGT supplementation was ascertained by our previous study [15]. The basal diet (Table 1) was formulated as recommended by the Ministry of Agriculture of China (NY/T 33–2004) to meet or exceed the requirements of broilers. The experiment lasted for 21 days. Throughout the entire experimental period, all the broilers were housed in a standard henhouse with ad libitum access to feed and water. The henhouse was cleaned, disinfected, and preheated to 35 °C in advance, ready for receiving the chicks. The room temperature was decreased by 0.5 °C per day except for the maintenance at 35 °C for the first week, regulated by a well-equipped ventilation system. Lighting began at 30–40 lx for 24 h during the first week and was reduced by 2 h weekly with 20 lx. The relative humidity was maintained at 60 ± 0.8% for the first week and then at 50 ± 0.8% for the subsequent three weeks. The person responsible for rearing the broilers was kept unaware of the group assignments.

### 2.2. LPS Challenge

On the 17th, 19th, and 21st days of the experiment (7:00 am), a total of eighteen broilers [one broiler from each replicate with a close body weight (BW) to the average] were selected from the CON, LPS, and LPS+CGT groups. The broilers from the LPS and LPS+CGT groups were intraperitoneally injected with 1 mg/kg BW *E. coli* O55:B5 LPS (L2880, Sigma, St. Louis, MO, USA), while the broilers from the CON group were intraperitoneally injected with an equivalent volume of 0.9% physiological saline, as described by Jiang et al. [24].

### 2.3. Sample Collection

On the 21st day of the experiment, 3 h after the injection of LPS or physiological saline, 10 mL of blood was collected from the wing vein of chickens in the three groups. The serum was obtained and stored at −20 °C after being centrifuged at 3000× *g* for 15 min at 4 °C. Following this, the chickens were euthanized by CO_2_ asphyxiation, and the small intestine was promptly separated following evisceration. Approximately 2 g of intestinal mucosa samples from the mid–small intestine were scraped and snap-frozen in liquid nitrogen after being placed into 2 mL of frozen storage tubes. Sections of around 2 cm in length from the mid-small intestine were cut and placed into a 4% paraformaldehyde solution for morphological observation. In addition, digesta from the caecum of each broiler was gathered into sterile bags. They were then stored at −80 °C until microbiological analysis was conducted. None of the people involved in the sample collection were informed of the group assignments in advance.

### 2.4. Measurements of Intestinal Morphology

After 24 h of fixation in a 4% paraformaldehyde solution, dehydration of the intestinal segments was performed in ethanol and xylene solutions, followed by embedding using a standard paraffin embedding protocol as described in a previous study [24]. The embedded tissue sections were chilled at 4 °C until the paraffin solidified. Subsequently, tissue sections of 5 μm in thickness were obtained using a Leica semi-automatic microtome (Leica CO., Wetzlar, Germany), put on slides, and dried at 37 °C for 24 h. After deparaffinage in xylene, a mixture of anhydrous ethanol and xylene and different concentrations of ethanol, samples on slides were stained with hematoxylin and eosin. Intestinal morphology was observed and photographed using a Nikon Eclipse 80i microscope (Nikon, Tokyo, Japan), and villus height (VH) and crypt depth (CD) were analyzed with JD801 software (version 1.0, JEDA, Nanjing, Jiangsu, China) according to the method described by Chen et al. [27]. The ratio of villus height to crypt depth (VH/CD) was calculated according to the values of VH and CD.

### 2.5. TUNEL Assay

The terminal deoxynucleotidyl transferase-mediated deoxyuridine triphosphate nick end labeling (TUNEL) test was performed in accordance with the manufacturer’s instructions to determine intestinal apoptosis using the TUNEL bright green apoptosis detection kit (A112, Jiancheng Bioengineering Institute, Nanjing, China). The stained images were viewed under an LSM 700 confocal laser scanning microscope (Carl Zeiss, Oberköchen, Germany) and used for cell counting. The TUNEL-positive cells (apoptotic cells) in square sampling regions (400 × 400 μm) were identified and counted for the calculation of positive cell density according to a previous study [24].

### 2.6. Determination of Intestinal Mucosal Caspase Activity and Total Protein Concentration

The intestinal mucosal activities of caspase-3 (C1115), caspase-8 (C1151), and caspase-9 (C1157) were quantified using ELISA kits purchased from Beyotime Biotechnology (Shanghai, China) according to the manufacturer’s instruction. In brief, 10 mg of intestinal mucosal tissue samples were homogenized using 100 μL of lysis buffer with a glass homogenizer, followed by being placed on an ice bath for 5 min. The supernatants of jejunal mucosal tissues were collected after centrifugation at 20,000× *g* for 10 min at 4 °C. Simultaneously, substrates of caspase-8 (Ac-IETD-*p*NA), caspase-9 (Ac-LEHD-*p*NA), and caspase-3 (Ac-DEVD-*p*NA) were pre-cooled. Subsequently, on the plate wells we added a 100 μL reaction mixture, which consisted of a 40 μL buffer solution, a 50 μL supernatant, and 20 μL of substrates. A wavelength of 405 nm was applied to measure the mixtures. Then 100 μL of the reaction mixture, which contained 20 μL of substrates, a 50 μL supernatant, and a 40 μL buffer solution, were added into the plate wells. The mixtures were incubated at 37 °C for 60 to 120 min, and their absorbance was then measured at 405 nm. In addition, Bradford Protein Assay Kit (Beyotime Biotechnology) was used for protein quantitation of the intestinal mucosal tissue samples. The activities of caspase-8, caspase-9, and caspase-3 in the intestinal mucosa have been standardized to the protein concentration per milligram in each sample.

### 2.7. Determination of Serum Diamine Oxidase (DAO), Intestinal Mucosal Barrier Factors, and Secretory Immunoglobulin A (sIgA) Concentrations

ELISA kits (Jiangsu Meimian Industrial Co., Ltd., Yancheng, China) were employed to measure serum DAO activity, and intestinal mucosal trefoil factor family (TFF), transforming growth factor-alpha (TGF-α), zonula occludens-1 (ZO-1), and sIgA concentrations. The testing steps for the indicators referred to a previous study by Chen et al. [28]. Briefly, around 0.1 g intestinal mucosa was homogenized in a 900 μL ice-cold saline solution. The supernatant was retained for the determination of intestinal mucosal barrier factors and sIgA after centrifuging at 12,000× *g* for 10 min at 4 °C. Then 50 μL prepared intestinal mucosa supernatant and serum were added into related microplates, as well as diluted standard solutions. Next, 100 μL HRP-Conjugated Reagent was added into each well, followed by a 30-min incubation at 37 °C. After washing five times with wash solution, a total of 100 μL of two kinds of color reagents were added to the wells. During the chromogenic procedure, the plates were incubated at 37 °C for 15 min, protected from light, and then the reaction was terminated by adding 50 μL stop buffer. Finally, the absorbance value of each well was subsequently measured at 450 nm. The concentrations of ZO-1, TFF, TGF-α, and sIgA in intestinal mucosa were normalized according to the total protein concentration of each sample.

### 2.8. Determination of Intestinal Mucosal Inflammatory Cytokines Concentrations

The determination of intestinal mucosal concentrations of inflammatory cytokines including interleukin-1β (IL-1β), interleukin-6 (IL-6), interleukin-10 (IL-10), tumor necrosis factor-α (TNF-α), and interferon-γ (IFN-γ) were performed with commercial ELISA kits obtained from R&D Systems Inc. (Minneapolis, MN, USA) with the manufacturer’s instruction as described briefly in Chen et al. [27]. After adding 100 μL of intestinal mucosal supernatant and standards, the microplates were incubated at room temperature for 2 h. After five washes, 100 μL of detection antibody was added to each well, and then the plates were incubated for another 2 h at room temperature. The plates were washed again and then incubated at room temperature for 30 min with the addition of 100 μL of two-color reagents. Finally, 100 μL of stop solution was added to each well. The optical density of each well was read at 450 nm.

### 2.9. Determination of the Intestinal Mucosal Malondialdehyde (MDA) Concentration

A commercial assay kit [thibabituric acid (TBA) method] purchased from Nanjing Jiancheng Bioengineering Institute (Nanjing, China) was applied to detect MDA concentration in the small intestinal mucosa. Solutions were added to the centrifuge tubes according to the instructions and thoroughly mixed. They were then subjected to a 95 °C water bath for 40 min. After cooled with running water, the samples were centrifuged at 3500 r/min for 10 min. The supernatant was collected to measure the absorbance value at 532 nm. Then the MDA content was normalized to the total protein concentration of each sample [27].

### 2.10. Determination of Relative mRNA Expression in the Intestinal Mucosa

The mucosal mRNA expression levels of the intestinal barrier genes [occludin (*OCLN*), claudin2 (*CLDN2*), claudin3 (*CLDN3*), and zonula occludens-1 (*ZO-1*)], nutrient transport-related genes [fatty acid binding protein-1 (*FABP1*), glucose transporter type 2 (*GLUT2*), Na+/glucose cotransporter (*SGLT1*), and y+L amino acid transporter 1 (*y+LAT1*)], inflammation-related genes [Toll-like receptor 4 (*TLR4*), myeloid differentiation primary response 88 (*MyD88*), and nuclear factor-kappa B (*NK-κB*)], antioxidant relative gene [catalase (*CAT*), glutathione peroxidase 1 (*GPX1*), nuclear factor erythroid 2-related factor 2 (*Nrf2*), heme oxygenase-1 (*HO-1*), superoxide dismutase 1 (*SOD1*), superoxide dismutase 2 (*SOD2*)] were accessed using real-time fluorescence quantitative PCR (qPCR). Total RNA was isolated from intestinal mucosa at 4 °C after being ground into powder in liquid nitrogen. Then the concentration of RNA was determined with NanoDrop2000 spectrophotometer (Thermo Fisher Scientific Inc., Waltham, MA, USA) and the integration and quality of RNA were examined by agarose gel electrophoresis. The concentration of RNA was adjusted to 100 ng/mL with RNase-free Water for reverse transcription according to Evo M-MLV Reverse Transcription Premix Kit (Accurate biology, Changsha, Hunan, China). The primer sequences (Supplementary Table S1) used for qPCR in this study, as well as the specific procedure for determining relative mRNA expression levels, were referenced in previous studies [15,16]. Briefly, the volume of reaction mixture, which contained SYBR, upstream primer, downstream primer and cDNA, was 10 µL. The qPCR protocol consisted of three steps. It commenced with denaturation at 95 °C for 300 s, followed by 35 cycles at 95 °C for 15 s, 60 °C for 15 s, and 72 °C for 15 s. Subsequently, a final step at 95 °C for 15 s, 65 °C for 60 s, and 97 °C for 15 s was performed. Each sample was determined for 3 times. The β-actin gene was amplified in parallel as an internal control for gene normalization and quantification. The relative mRNA abundance of the target genes in the intestinal mucosal samples was then calculated using 2^−ΔΔCt^ method [29].

### 2.11. Cecal Microbial Composition and Diversity Analysis

Following the manufacturer’s instructions, the E.Z.N.A.^TM^ fecal DNA kit (Omega Bio-Tek, Norcross, GA, USA) was used to extract bacterial genomic DNA from the frozen cecal contents. The concentration and purity of the extracted DNA was detected using a 1% agarose gel. Subsequently, sterile water was used to dilute DNA to a concentration of 1 ng/μL. The detection of the DNA concentration and purity was detected by a 1% agarose gel for DNA and then diluted with sterile water to 1 ng/μL. The 16S rDNA V4 hypervariable region was amplified using 515F and 806R primers (5′-GTGCCAGCMGCCGCGGTAA-3′ and 5′-GGACTACHVGGGTWTCTAAT-3′, respectively) [30], and a Qiagen gel extraction kit (Qiagen, Germany) was applied to purify the PCR product. Subsequently, the sequencing library was created and its quality was assessed, followed by 250 bp paired-end reads generation. After end-to-end read assembly, the next step involved data filtering. Subsequently, chimera removal was performed to obtain effective sequences. UPARSE (version 7.0.1001) software was used for a high-quality sequences clustering analysis, and the operational taxonomic units (OTUs) were formed by clustering sequences sharing over 97% similarity. The SILVA 138 database was then used for classifying these OUTs at different taxonomic levels [30]. Then, MUSCLE (version 3.8.31) was used for rapid multiple sequence alignment followed by data standardization. Alpha diversity (observed species, Shannon index, Simpson index, Chao 1 index, and ACE index) and beta diversity based on Bray–Curtis distance were performed for comparison of taxonomic data in the Quantitative Insights Into Microbial Ecology version 2 (QIIME 2) [27]. The plots were generated using R software (version 2.15.3). The assessment of dissimilarities within microbial communities was conducted using the analysis of similarity (ANOSIM) test to identify significant variations. Functional Annotation of Prokaryotic Taxa (FAPROTAX) was used to obtain functional annotation information used for functional prediction [31].

### 2.12. Statistical Analysis

An individual broiler was considered as the experimental unit, and Shapiro–Wilk test was used to check the data normality (W > 0.05). Then the data were subjected to statistical analysis using ANOVA to evaluate the statistically significant differences among the three groups with SAS 9.4 software. Meanwhile, the least significant difference (LSD) method was performed to compare the differences among the groups. The data were expressed as means ± standard error. A level of *p* < 0.05 was considered as significantly different, while 0.05 < *p* < 0.10 was considered as a trend towards significance.

## 3. Results

### 3.1. Intestinal Morphology

**Figure 1:**
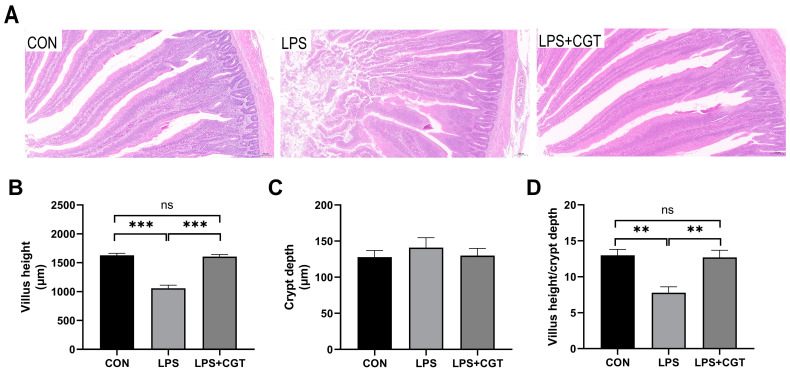
Effects of Chinese gallotannins (CGT) supplemented in the diet on intestine histolomorph of broilers exposed to lipopolysaccharide (LPS). (**A**) Photomicrographs obtained at 100× magnification stained by hematoxylin and eosin; (**B**) villus height; (**C**) crypt depth; (**D**) villus height/crypt depth. CON, broilers fed a basal diet and administrated saline solution intraperitoneally; LPS, broilers fed a basal diet and administrated LPS intraperitoneally; LPS+CGT, broilers fed a basal diet with 300 mg/kg CGT supplementation and administrated LPS intraperitoneally. Data are presented as means with standard error of the mean (SEM, *n* = 6). Significant differences between groups are indicated by ** *p* < 0.01 and *** *p* < 0.001. Non-significant differences are indicated by “ns”.

The effects of dietary CGT supplementation on the intestinal morphology in broilers challenged with LPS are shown in Figure 1. LPS administration evidently disrupted the regularity and order of the intestinal villi, and led to villus shedding and atrophy, while dietary CGT addition attenuated the undesirable changes in the intestinal mucosa induced by the LPS challenge (Figure 1A). Moreover, broilers in the LPS group had significantly lower VH (Figure 1B) and VH/CD ratio (Figure 1D) compared with broilers in the CON and LPS+CGT groups (*p* < 0.05), while VH and VH/CD ratio in the CON and LPS+CGT groups did not significantly differ (*p* > 0.05). No significant difference was observed in CD (Figure 1C) among the three groups (*p* > 0.05).

### 3.2. Intestinal Cell Apoptosis and Caspase Activity

**Figure 2:**
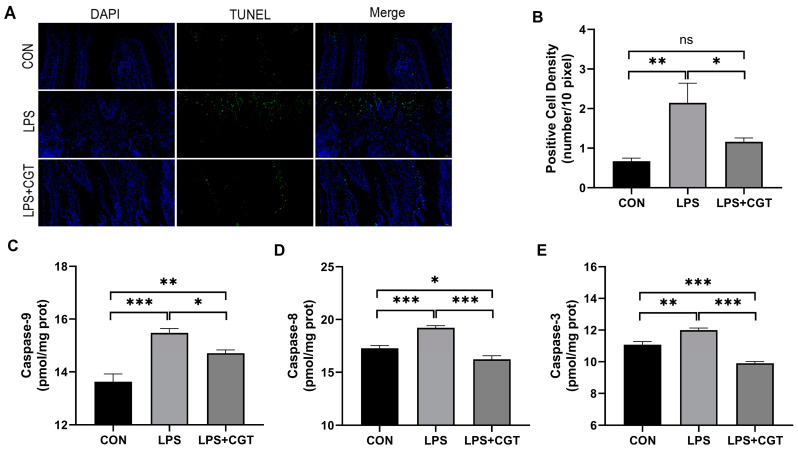
Effects of Chinese gallotannins (CGT) supplemented in the diet on intestine cell apoptosis and caspase activity in broilers challenged with lipopolysaccharide (LPS). (**A**) TUNEL assay of small intestinal sections of LPS-challenged broilers by immunofluorescence, in which blue represents the total number of cells while green represents the apoptotic cells of the small intestine; (**B**) positive cell density; (**C**) caspase-9; (**D**) caspase-8; (**E**) caspase-3. CON, broilers fed a basal diet and administrated saline solution intraperitoneally; LPS, broilers fed a basal diet and administrated LPS intraperitoneally; LPS+CGT, broilers fed a basal diet with 300 mg/kg CGT supplementation and administrated LPS intraperitoneally. Data are presented as means with standard error of the mean (SEM, *n* = 6). Significant differences between groups are indicated by * *p* < 0.05 ** *p* < 0.01 and *** *p* < 0.001. Non-significant differences are denoted by “ns”.

The results of the TUNEL assay showed that the apoptosis (positive) cells were distributed mainly in the apical region of the small intestinal villi (Figure 2A), and the positive cell density significantly increased (Figure 2B) in LPS broilers in comparison to the CON broilers and LPS+CGT broilers (*p* < 0.05). Meanwhile, compared with the CON broilers, LPS broilers exhibited significantly increased intestinal mucosal caspase-9 (Figure 2C), caspase-8 (Figure 2D), and caspase-3 (Figure 2E) activities (*p* < 0.05); mucosal caspase-9, caspase-8, and caspase-3 activities were significantly reduced in LPS-challenged broilers supplemented with CGT (*p* < 0.05). Furthermore, the broilers in the LPS+CGT group had significantly higher caspase-9 activity (*p* < 0.05) with significantly lower caspase-8 and caspase-3 activities (*p* < 0.05) in the intestinal mucosa compared with the broilers in the CON group.

### 3.3. Maturity and Integrity of the Intestinal Mucosal Barrier

**Figure 3:**
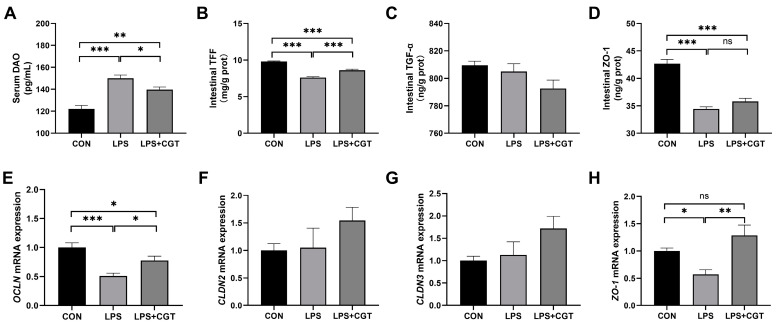
Effects of Chinese gallotannins (CGT) supplemented in the diet on the maturity and integrity of the intestinal mucosal barrier in broilers with lipopolysaccharide (LPS) challenge. (**A**) Serum DAO, serum diamine oxidase; (**B**) intestinal TFF, trefoil factor family; (**C**) intestinal TGF-α, transforming growth factor-α; (**D**) intestinal ZO-1, zonula occludens-1; (**E**) *OCLN*, occludin; (**F**) *CLDN2*, claudin-2; (**G**) *CLDN3*, claudin-3; (**H**) *ZO-1*, zonula occludens-1. CON, broilers fed a basal diet and administrated saline solution intraperitoneally; LPS, broilers fed a basal diet and administrated LPS intraperitoneally; LPS+CGT, broilers fed a basal diet with 300 mg/kg CGT supplementation and administrated LPS intraperitoneally. Data are presented as means with standard error of the mean (SEM, *n* = 6). Significant differences between groups are indicated by * *p* < 0.05, ** *p* < 0.01 and *** *p* < 0.001. Non-significant differences are denoted by “ns”.

Figure 3 shows the effects on the maturation and integrity of the intestinal mucosal barrier in LPS-challenged broilers supplemented with CGT. The LPS challenge significantly increased serum DAO activity (Figure 3A) (*p* < 0.05), while significantly decreasing TFF (Figure 3B) and ZO-1 (Figure 3D) concentrations alongside *OCLN* (Figure 3E) and *ZO-1* (Figure 3H) mRNA expressions in the intestinal mucosa (*p* < 0.05). Dietary CGT supplementation significantly decreased serum DAO activity (*p* < 0.05), and significantly increased intestinal TFF content, as well as *OCLN* and *ZO-1* mRNA expressions, in LPS-challenged broilers (*p* < 0.05). Nevertheless, the broilers in the LPS+CGT group exhibited significantly elevated serum DAO activity (*p* < 0.05) and significantly lower TFF and ZO-1 contents and *OCLN* mRNA expression (*p* < 0.05) compared with the broilers of the CON group. There were no significant differences observed in TGF-α (Figure 3C) content and *CLDN2* (Figure 3F) and *CLDN3* (Figure 3G) mRNA expressions in the intestinal mucosa among the three groups (*p* < 0.05).

### 3.4. Intestinal Nutrient Transport-Related Gene Expression

**Figure 4:**
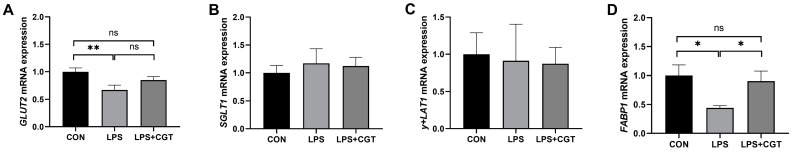
Effects of Chinese gallotannins (CGT) supplemented in the diet on the relative expression of genes related to intestinal nutrient transport in broilers injected with lipopolysaccharide (LPS). (**A**) *GLUT2*, glucose transporter type 2; (**B**) *SGLT1*, Na+/glucose cotransporter; (**C**) *y + LAT1*, y + L amino acid transporter 1; (**D**) *FABP1*, fatty acid binding protein-1. CON, broilers fed a basal diet with no challenge; LPS, broilers fed a basal diet and challenged by LPS intraperitoneally; LPS+CGT, broilers fed a basal diet with 300 mg/kg CGT supplementation and challenged by LPS intraperitoneally. Data are presented as means with standard error of the mean (SEM, *n* = 6). Significant differences between groups are indicated by * *p* < 0.05 and ** *p* < 0.01. Non-significant differences are denoted by “ns”.

The effects of dietary CGT supplementation on the expression of intestinal nutrient transport-related genes in broilers challenged with LPS are shown in Figure 4. Compared with the CON group, LPS injection significantly decreased the mRNA expression of *GLUT2* (Figure 4A) and *FABP1* (Figure 4D) (*p* < 0.05). In contrast, dietary CGT supplementation attenuated the LPS-induced decreases in mRNA expressions of *GLUT2* and *FABP1* to levels observed in the CON group (*p* > 0.05). No significant differences were observed in mRNA expression of *SGLT1* (Figure 4B) and *y + LAT* (Figure 4C) were observed among the three groups (*p* > 0.05).

### 3.5. Intestinal Inflammatory Factors and Inflammation-Related Gene Expressions

**Figure 5:**
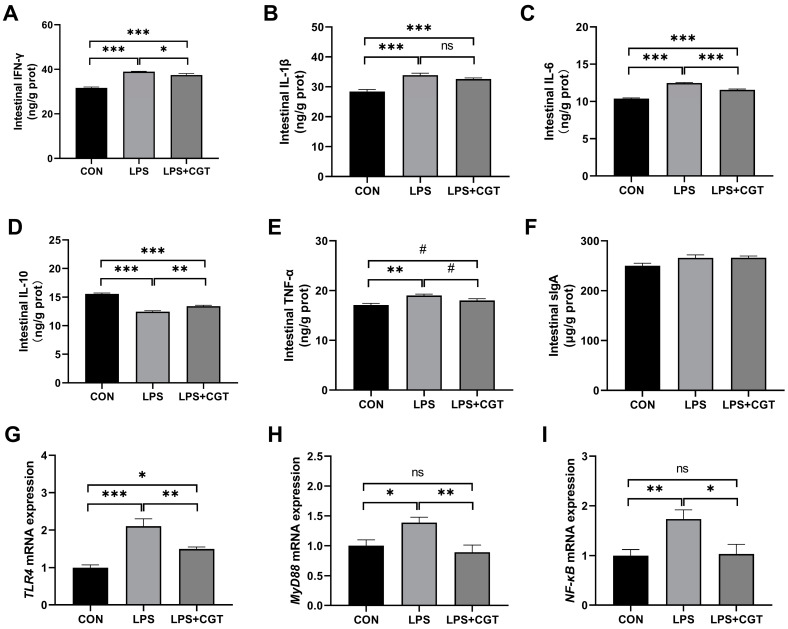
Effects of dietary Chinese gallotannins (CGT) supplementation on intestinal inflammatory factors and the expression of inflammation-related genes in broilers challenged with lipopolysaccharide (LPS). (**A**) Intestinal IFN-γ, interferon-γ; (**B**) intestinal IL-1β, interleukin-1β; (**C**) intestinal IL-6, interleukin-6; (**D**) intestinal IL-10, interleukin-10; (**E**) intestinal TNF-α, tumor necrosis factor-α; (**F**) intestinal sIgA, secretory Immunoglobulin-A; (**G**) *TLR4*, Toll-like receptor 4; (**H**) *MyD88*, myeloid differentiation primary response protein 88; (**I**) *NF-κB*, nuclear factor-κ-gene binding. CON, broilers fed a basal diet with no challenge; LPS, broilers fed a basal diet and challenged by LPS intraperitoneally; LPS+CGT, broilers fed a basal diet with 300 mg/kg CGT supplementation and challenged by LPS intraperitoneally. Data are presented as means with standard error of the mean (SEM, *n* = 6). Significant differences between groups are indicated by * *p* < 0.05, ** *p* < 0.01 and *** *p* < 0.001. A trend towards significance was displayed as # 0.05 < *p* < 0.01. Non-significant differences are denoted by “ns”.

As shown in Figure 5, the LPS challenge significantly increased intestinal mucosal IFN-γ (Figure 5A), IL-1β (Figure 5B), IL-6 (Figure 5C), and TNF-α (Figure 5E) concentrations (*p* < 0.05), while significantly decreasing the IL-10 (Figure 5D) concentration compared to the CON group (*p* < 0.05). In LPS-challenged broilers, CGT supplementation significantly increased IL-10 level (*p* < 0.05), significantly decreased IFN-γ, IL-1β, and IL-6 levels (*p* < 0.05) and tended to decrease the TNF-α concentration (*p* < 0.10) in the intestinal mucosa. Moreover, compared with CON broilers, the broilers in LPS+CGT group showed significantly increased IFN-γ, IL-1β, IL-6, and IL-10 contents (*p* < 0.05), and significantly reduced IL-10 concentration (*p* < 0.05) in the intestinal mucosa, while also tending to have a decreased intestinal mucosal TNF-α content (*p* < 0.10). No significant difference was observed in intestinal mucosal sIgA (Figure 5F) concentration among the three groups (*p* > 0.05).

The genes expressions results show that compared to the CON group, LPS administration resulted in a significant increase in the mRNA expression of *TLR4* (Figure 5G), *MyD88* (Figure 5H), and *NF-κB* (Figure 5I) (*p* < 0.05). Dietary CGT supplementation significantly suppressed intestinal mucosal *TLR4*, *MyD88*, and *NF-κB* mRNA expression compared with the LPS broilers (*p* < 0.05). However, significantly higher *TLR4* mRNA expression was observed in the LPS+CGT broilers than in the CON broilers (*p* < 0.05).

### 3.6. Intestinal MDA Concentration and Antioxidant-Related Gene Expression

**Figure 6:**
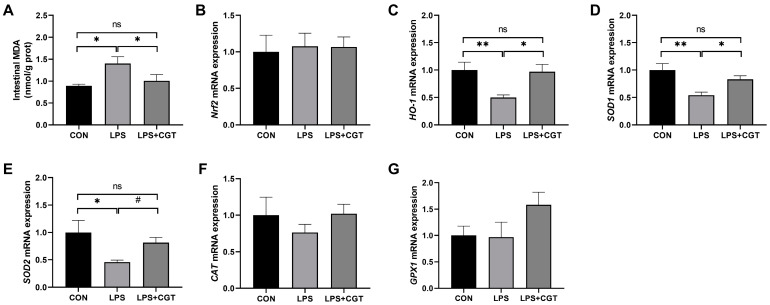
Effects of Chinese gallotannins (CGT) supplemented in diet on intestinal malonaldehyde concentration and the expression of antioxidant-related genes in broilers challenged with lipopolysaccharide (LPS). (**A**) Intestinal MDA, malonaldehyde; (**B**) *Nrf2*, nuclear factor erythroid 2-related factor 2; (**C**) *HO-1*, heme oxygenase-1; (**D**) *SOD1*, superoxide dismutase 1; (**E**) *SOD2*, superoxide dismutase 2; (**F**) *CAT*, catalase; (**G**) *GPX1*, glutathione peroxidase 1. CON, broilers fed a basal diet and administrated saline solution intraperitoneally; LPS, broilers fed a basal diet and administrated LPS intraperitoneally; LPS+CGT, broilers fed a basal diet with 300 mg/kg CGT supplementation and administrated LPS intraperitoneally. Data are presented as means with standard error of the mean (SEM, *n* = 6). Significant differences between groups are indicated by * *p* < 0.05 and ** *p* < 0.01. A trend towards significance was displayed as # 0.05 < *p* < 0.01. Non-significant differences are denoted by “ns”.

As exhibited in Figure 6, LPS injection significantly increased MDA (Figure 6A) concentration (*p* < 0.05), and significantly decreased *HO-1* (Figure 6C), *SOD1* (Figure 6D), and *SOD2* (Figure 6E) mRNA expressions (*p* < 0.05) in the intestinal mucosa compared with the CON group. In contrast, dietary CGT supplementation attenuated LPS-induced changes in the MDA concentration (*p* < 0.05) and *HO-1* (*p* < 0.05), *SOD1* (*p* < 0.05), and *SOD2* (*p* < 0.10) mRNA expression to levels observed in the CON group. Both LPS-challenge and CGT supplementation didn’t alter the relative mRNA expressions of *Nrf2* (Figure 6B), *CAT* (Figure 6F), and *GPX1* (Figure 6G) (*p* > 0.05).

### 3.7. Microbial Composition and Diversity

#### 3.7.1. Bacterial Community Diversity and Richness

**Figure 7:**
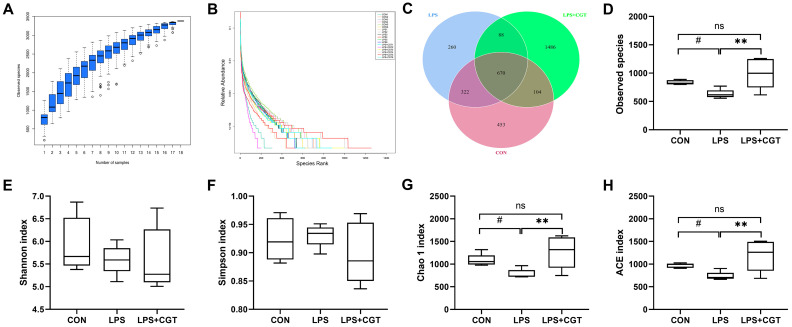
Differences in the diversity and richness of bacterial community among the three groups. (**A**) Species accumulation curve; (**B**) Rank abundance curve reflecting species richness by the width of the curve in the horizontal direction and species evenness by the smoothness of the curve in the vertical direction; (**C**) A Venn diagram of operational taxonomic units; (**D**) Observed species; (**E**) Shannon index; (**F**) Simpson index; (**G**) Chao1 index; (**H**) ACE index. CON, broilers fed a basal diet and administrated saline solution intraperitoneally; LPS, broilers fed a basal diet and administrated LPS intraperitoneally; LPS+CGT, broilers fed a basal diet with 300 mg/kg CGT supplementation and administrated LPS intraperitoneally. *n* = 6. ** *p* < 0.01 indicates significant differences between groups. A trend towards significance was displayed as # 0.05 < *p* < 0.01. Non-significant differences are denoted by “ns”.

As shown in Figure 7A, with the number of analyzed samples rising to 18, species accumulation curves tended to be flat, indicating that our samples were sufficient for data analysis and species richness prediction. The distribution of bacterial taxa abundance was plotted as rank abundance curves (Figure 7B) to assess the differences in bacterial richness and evenness, suggesting that the LPS+CGT group had the greatest species richness and homogeneous species distribution compared with other groups in the order: LPS+CGT > CON > LPS. Consistently, as the Venn diagram (Figure 7C) displayed, the number of unique OTUs was greatest (1486) in the LPS+CGT group and smallest (260) in the LPS group. For the alpha diversity indices, LPS administration tended to decrease the observed species (Figure 7D), Chao 1 index (Figure 7G), and ACE index (Figure 7H) compared with the CON group (*p* < 0.10); however, dietary CGT supplementation significantly prevented LPS-induced decreases in these alpha diversity indices, resulting in similar levels as those observed in the CON group (*p* < 0.05). There were no significant differences among the three groups in the Shannon (Figure 7E) and Simpson (Figure 7F) indices (*p* > 0.05).

#### 3.7.2. Beta Diversity of Microbial Community Analysis

**Figure 8:**
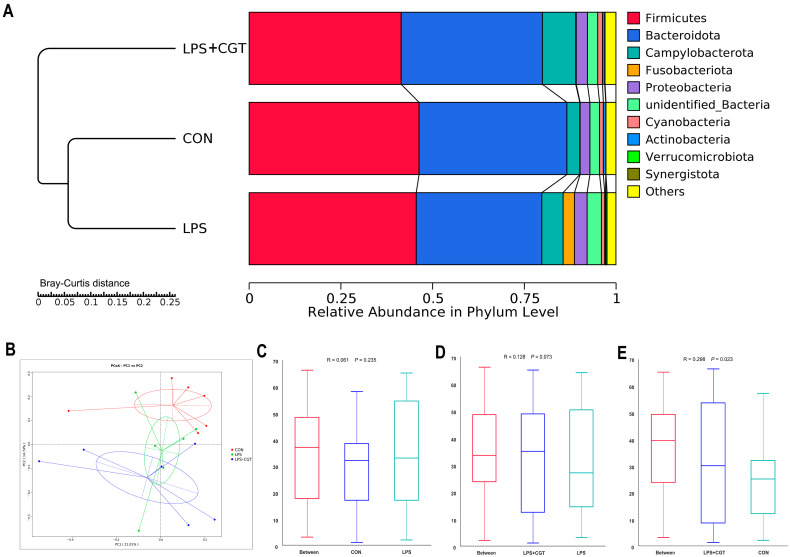
Beta diversity of microbial communities. (**A**) Unweighted pair group method with arithmetic mean (UPGMA) clustering analysis using Bray–Curtis distance; (**B**) The principal coordinate analysis (PCoA) profile based on Bray–Curtis distance; (**C**–**E**) Similarity analysis (CON vs. LPS, CGT vs. LPS, and LPS + CGT vs. CON). The value of R is with the range of −1 to +l. Typically, 0 < R < 1 and *p* < 0.05 indicates the significant differences between the groups. CON, broilers fed a basal diet and administrated saline solution intraperitoneally; LPS, broilers fed a basal diet and administrated LPS intraperitoneally; LPS+CGT, broilers fed a basal diet with 300 mg/kg CGT supplementation and administrated LPS intraperitoneally. *n* = 6.

The unweighted pair-group method with arithmetic means (UPGMA) phylogenetic tree based on Bray–Curtis distance at the phylum level is displayed in Figure 8A. When clustered, the CON and LPS groups showed a closer distance and greater similarity. In contrast, the LPS+CGT group formed a separate branch, distinct from the other two groups. The principal coordinate analysis (PCoA) (Figure 8B) combined with the ANOSIM analysis (Figure 8C–E) also indicated that the bacterial community structures in the LPS+CGT and CON groups were significantly different (Figure 8E) (*p* < 0.05), and the bacterial community structures between the LPS+CGT and LPS groups had a trend toward significant difference (Figure 8D) (*p* < 0.10). The bacterial community structure between the CON and LPS groups was similar (Figure 8C) (*p* > 0.05).

#### 3.7.3. Microbial Relative Abundance at the Phylum and Genus Levels

As shown in Figure 8A, Firmicutes and Bacteroidetes were the two most plentiful phyla in all three groups, accounting for, on average, 44.43% and 37.67% per sample, respectively. However, we did not observe significant differences in the relative abundance of the top 10 phyla of the cecal microbiota in broilers (*p* > 0.05).

**Figure 9:**
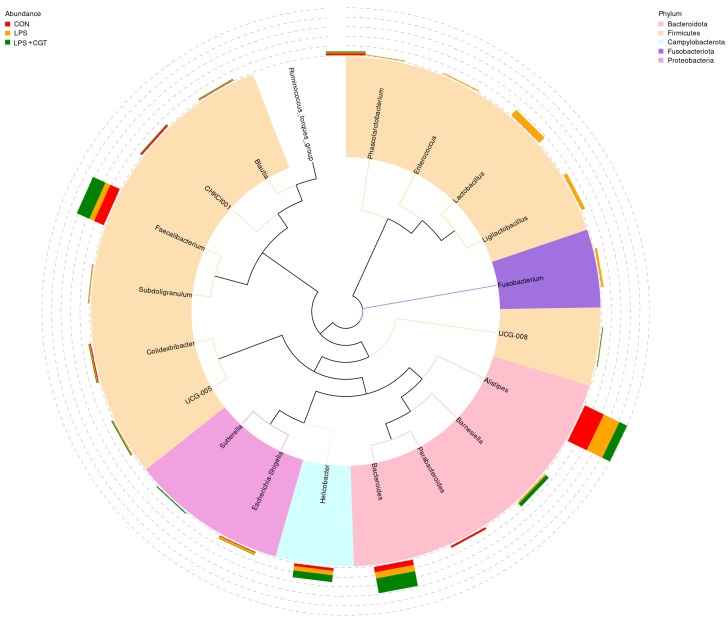
A phylogenetic tree built with the top 20 most abundant genera. The branches in the inner circle are color-coded to represent the corresponding phylum. In the outer circle, a stacked column chart indicates the relative abundance of each genus across different treatments. CON, no CGT treatment or LPS challenge; LPS, LPS challenge + 0 mg/kg CGT; LPS+CGT, LPS challenge + 300 mg/kg CGT. *n* = 6.

The relative abundances of the top 20 genera in the cecal microbiota of broilers in broilers are illustrated in the phylogenetic tree (Figure 9) and detailed in Supplementary Table S2. The dominant genera for each treatment are displayed individually: in the CON group, Bacteroidota comprised 27.61% Alistipes and 7.19% Bacteroides, and Firmicutes contained 13.61% Faecalibacterium; in the LPS group, Firmicutes included 8.86% *Lactobacillus*, 6.98% *Faecalibacterium*, and 4.67% *Ligilactobacillus*, and Bacteroidota contained 22.58% *Alistipes* and 8.22% *Bacteroides*; in the LPS+CGT group, Firmicutes included 17.91% *Faecalibacterium*, Bacteroidota was composed of 19.66% *Bacteroides*, 11.14% *Alistipes*, and 5.05% *Barnesiella*, while Campylobacterota contained *Helicobacter*. Moreover, there was a trend towards reduced Alistipes abundance in the LPS+CGT group compared with the CON group (*p* < 0.10).

#### 3.7.4. Predicted Function and Characteristics of Cecal Microbiota

**Figure 10:**
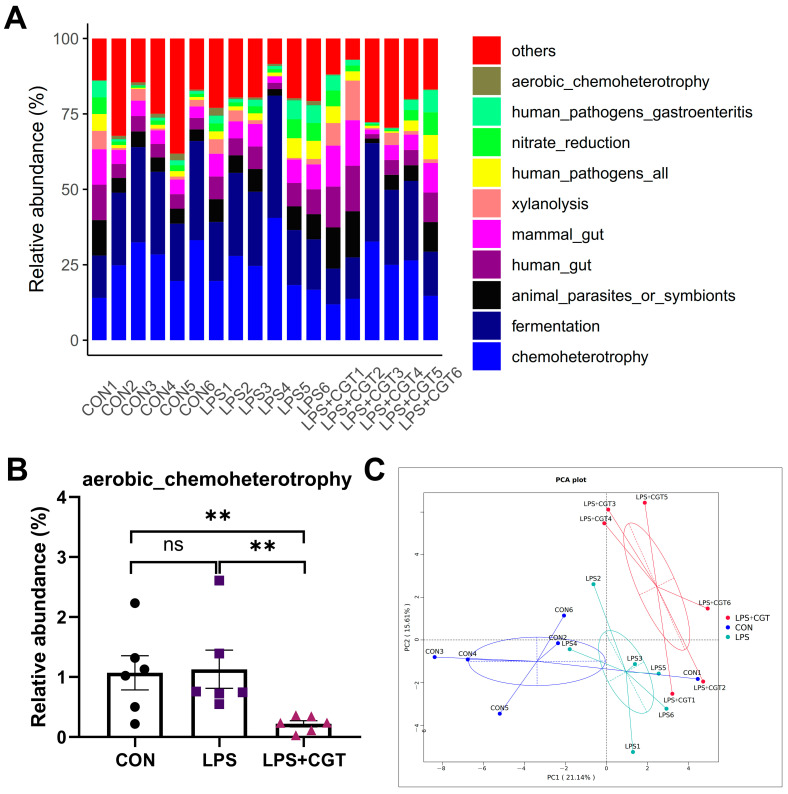
Relative abundance of the predicted functions of the cecal microbiota among the three groups. (**A**) Functional relative abundance histogram formed based on the functional information of each group in the top 10 of the maximum abundance at each annotation level; (**B**) Relative abundance of aerobic_chemoheterotrophy (Values are means ± standard error); (**C**) Principal component analysis (PCA) profile. CON, no CGT treatment or LPS challenge; LPS, LPS challenge + 0 mg/kg CGT; LPS+CGT, LPS challenge + 300 mg/kg CGT. Data are presented as means with standard error (SEM, *n* = 6). Significant differences between groups were indicated by ** *p* < 0.01. Non-significant differences are denoted by “ns”.

The relative abundance of the top 10 predicted microbial functions by FAPROTAX is shown in Figure 10A. Of the top 10 functions, chemoheterotrophy and fermentation were the top two functional annotations in the three groups, and the relative abundance of aerobic_chemoheterotrophy was significantly lower in the LPS+CGT group than in the CON and LPS groups (Figure 10B) (*p* < 0.05). Moreover, principal component analysis (Figure 10C) revealed that the microbial function composition in the LPS+CGT group was distinctly separate from the CON and LPS groups, with dispersion between the LPS+CGT group and the CON and LPS groups.

## 4. Discussion

The intestinal tract is an important digestive and absorptive organ, and efficient uptake of nutrients relies on the crypt–villus structural integrity [4,5]. The VH and VH/CD are two crucial indicators for evaluating intestinal health and absorption capacity [24,32]. Small intestinal villi can effectively increase the absorption area of the intestinal mucosa, and increased VH results in a larger intestinal absorption area and stronger absorption capacity [27]. Consistent with prior studies, LPS challenge severely damaged the intestinal morphology, causing obvious breakage and erosion of broiler intestinal villi, and decreased VH and VH/CD ratio [24,27]. Studies on different animal models demonstrated that tannins can enhance intestinal structure and function. Xu et al. [18] reported that 500, 750, and 1000 mg/kg tannins supplementations increased the ileal VH in necrotic enteritis-infected broilers. Previous work in piglets also found that adding 0.2% tannins to the diet decreased CD and increased VH/CD ratio in the duodenum and ileum [33]. Moreover, Wang et al. [34] indicated that the dose of 2.5 mg kg^−1^ tannins ameliorated diquat-induced morphological damage in the jejunum via increasing the VH in mice. Taken together, our findings suggested that the dietary addition of CGT could protect the intestinal morphology against the damage caused by LPS challenge, which is crucial for nutrient absorption and overall health. Furthermore, we also observed changes in mRNA expressions of nutrient transporters, including *GLUT2* and *FABP1*. *GLUT2* acts as a glucose sensor catalyzing glucose transport across the plasma membrane, and its inactivation results in limited glucose uptake [35]. *FABP1* encapsulates a variety of hydrophobic ligands, such as long-chain fatty acids and fatty acyl coenzyme A, which are important for the metabolism and transportation of fatty acids [36]. In the current study, we found that 300 mg/kg CGT supplementation inhibited the reduction in *GLUT2* and *FABP1* mRNA expression caused by the LPS challenge, suggesting that dietary CGT supplementation promoted the intestinal absorption of glucose and fatty acids under LPS challenge in broilers. Xu et al. [18] demonstrated that dietary 500 mg/kg tannin supplementation relieved the necrotic enteritis infection-induced decrease in glucose transporter *SGLT1* expression in the ileum of broilers. Previous research in piglets has indicated that adding 1000 mg/kg tannins to the diet can up-regulate the gene expression of amino acid transporter *B^0^AT1* and peptide transporter *PepT1* in the ileum [37]. To summarize, dietary 300 mg/kg CGT supplementation conveyed benefits in intestinal development and absorption function in LPS-challenged broilers.

Besides the nutrient absorption function, the intestine also serves as a physical barrier against the invasion of harmful microorganisms and pathogens. Increased intestinal permeability can lead to systemic inflammation and an increased susceptibility to infections. Serum DAO activity is considered as a dependable indicator of intestinal barrier function [24,38]. DAO is an intracellular enzyme in the intestinal epithelium. When the permeability of the intestine increases abnormally due to a disruption, DAO from the lumen readily crosses the intestinal mucosa and enters the peripheral bloodstream [24]. The intestinal barrier function is regulated by tight junctions composed of transmembrane proteins and cytoplasmic scaffold proteins, including CLDN, OCLN, and ZO-1 [39]. The potent growth factor TFF can regenerate and repair damaged gastrointestinal mucosa, protecting the gastrointestinal mucosa against injury [40]. Therefore, LPS administration may destroy the intestinal barrier function by inhibiting the expression of ZO-1 and OCLN and the generation of TFF in the current study. However, the related pathway of up-regulating TFF and tight junction proteins requires further investigation. LPS injection damaged the small intestinal barrier function and increased serum DAO activity in broilers [24]. Tannin supplementation can enhance the intestinal barrier and alleviate increased intestinal permeability. Xu et al. [18] found that supplementing the diet with three different levels of tannins elevated the mRNA expression of *ZO-1*, *MUC2*, and *OCLN*, respectively, in the ileum in necrotic enteritis-infected broilers after a 28-d treatment. The research in piglets by Yu et al. [33] also showed that dietary tannins supplementation increased the *OCLN*, *ZO-1*, and *ZO-2* expression, and reduced serum DAO activity. Therefore, our findings indicate that 300 mg/kg dietary CGT supplementation partially inhibited the intestinal barrier function damage caused by LPS challenge in broilers. Compared with the CON group, there was no difference in the mRNA expression of *ZO-1*, while the content of ZO-1 protein was significantly lower in the LPS+CGT group. Protein synthesis is regulated by genes, but influenced by various factors during transcription, translation, and post-translational modifications. The specific reason behind this phenomenon requires further investigation.

Microorganisms are located on the surface of the intestinal mucosa, and constitute the intestinal barrier together with the mucus layer, epithelium, and mucosal immune system [41,42]. Once the intestinal barrier function becomes damaged, it will also cause the disruption of intestinal microbiota, leading to diarrhea, growth retardation, and even death in poultry production [38,43]. In the current study, dietary CGT supplementation reversed LPS-induced decreases in the alpha diversity indices (including observed species, Chao1, and ACE) of the cecal microbiota, indicating that CGT supplementation increased the unique OTUs and community richness in LPS-challenged broilers. A lower alpha diversity is usually associated with dysbacteriosis [44]. Furthermore, the LPS+CGT group showed a different bacterial community structure and predicted functions compared with the CON and LPS groups. Interestingly, the cecal bacterial structure of the CON group and LPS group exhibited a similar bacterial community in this study, which was aligned with the results in Chen et al. [45]. The possible reason for this outcome may be that the dietary treatment was the main factor causing microbial changes in this experiment, while the duration of the LPS challenge was too short to cause significant alterations in the bacterial community structure [27]. In particular, the predicted function aerobic_chemoheterotrophy was lower in the LPS+CGT group compared to the CON and LPS groups. The intestinal cavity is an anaerobic environment, dominated by anaerobic microorganisms [27,29]. The inflammatory response is known to increase the oxygen content in the intestinal lumen, thus promoting the presence of harmful aerobic microorganisms (such as *Escherichia-Shigella* and *Streptococcus*) and reducing the number of beneficial obligate anaerobes, including *Bifidobacterium* and *Lactobacillus* [31,46]. The alterations in intestinal bacterial function indicated that the addition of CGT was beneficial for inhibiting the proliferation of harmful bacteria [47]. Therefore, dietary supplementation with 300 mg/kg CGT could maintain intestinal microbiota homeostasis through alleviating LPS-induced reductions in alpha diversity and inhibiting the function of aerobic microorganisms in broilers. Nevertheless, the correlation between gut bacteria and CGT in maintaining intestinal health requires further investigation.

Apoptosis is a kind of programmed cell death controlled by the expression of genes to eliminate unwanted or harmful cells and reshape tissue to maintain normal physiological functions [48]. Excessive enterocytic apoptosis is a driving factor in the intestinal barrier damage induced by LPS challenge [27], which will lead to the failure of intestinal epithelial cells to update and repair structural damage in time [49]. TUNEL staining in our study demonstrated that enterocyte apoptosis induced by LPS can be suppressed by CGT, primarily in the apical region of the small intestinal villi, thus protecting the intestinal barrier integrity. The apoptosis pathway is divided into intrinsic and extrinsic apoptosis, with the common characteristic of apoptosis being the activation of caspases. The occurrence of extrinsic apoptosis depends on the activation of caspase-8, while caspase-9 is a key component of the intrinsic pathway. Caspase-8 and caspase-9 can both activate the effector caspase-3, thereby initiating the process of apoptosis [50]. In the present study, the LPS challenge activated caspase-8, caspase-9, and caspase-3, which was consistent with the results from Li et al. [51]. Inversely, dietary CGT supplementation suppressed the elevation of these caspase activities. Niu et al. [13] also demonstrated that dietary addition of CGT reduced the hepatic activity of caspase-3 in broilers. Similarly, a previous study in mice indicated that tannins had a significant reduction effect on arsenic trioxide-induced caspase-3 elevation in the heart [52]. Therefore, our study suggested that a 300 mg/kg CGT supplementation could suppress LPS-induced intestinal mucosal injury via inhibition of caspase-associated apoptosis, which helps in maintaining the integrity of the intestinal barrier and overall intestinal health.

The inflammatory response is another critical factor influencing intestinal health, which can induce enterocyte apoptosis and intestinal barrier damage [27]. The TLR4/NF-κB pathway plays a crucial role in immune and inflammatory responses [53]. TLR4, an LPS recognition receptor, combines with the LPS, and thereby activates NF-κB via the MyD88 protein, causing the release of inflammatory cytokines [53,54]. Previous research in broilers by Huang et al. [55] implied that LPS administration up-regulated the expression of TLR4 and its downstream molecules (MyD88, NF-κB, TNF-α and IL-1β) in the chicken liver. The tannins have been proven to suppress the activation of the TLR4 signaling pathway and the release of pro-inflammatory cytokines (TNF-α, IL-1β, IL-6, and IFN-γ) [43]. Consistently, we found that dietary CGT supplementation inhibited intestinal inflammatory responses in LPS-challenged broilers by decreasing IL-6 and TNF-α concentrations and increasing IL-10 concentration, as well as the expression of genes related to the TLR4/NF-κB pathway in the intestinal mucosa. Similarly, Song et al. [43] showed that CGT could attenuate enterotoxigenic *E. coli* O101 infection-induced increases in serum concentrations of pro-inflammatory cytokines including IFN-γ, TNF-α, IL-1β, IL-6, and IL-8 in mice. Taken together, a 300 mg/kg dietary CGT supplementation could suppress the release of inflammatory cytokines by inhibiting the TLR4/NF-κB pathway, ultimately decreasing the inflammatory responses of the intestinal mucosa, which is crucial for maintaining the intestinal health of broiler chickens [53].

Intestinal inflammatory responses are often accompanied by the occurrence of oxidative stress, which may exacerbate intestinal mucosal barrier dysfunction [5,24]. When pathogenic bacteria attack the intestinal mucosa, the balance between reactive oxygen species production and the antioxidant defense capacity of the body will be disrupted [56]. It has been proven that intraperitoneal LPS injection can result in intestinal inflammation and oxidative stress, concurrently [24,55]. Consistently, in the present study, LPS challenge elevated the MDA concentration and reduced the relative mRNA expressions of *HO-1*, *SOD1*, and *SOD2* in the intestinal mucosa, which was mitigated to levels observed in the CON group by dietary CGT supplementation. MDA is the byproduct of lipid oxidation, and its level reflects the degree of peroxidation [16]. Lipid peroxidation can lead to cell death and produce toxic free radicals. HO-1, an inducible heme degradation enzyme, provides a cytoprotective function against diverse oxidative and inflammatory stress, as well as cell apoptosis [16,57], while SOD catalyzes the conversion of superoxide anions into relatively less toxic hydrogen peroxide [24]. Tannins exhibit effective antioxidant effects across various species. Yuan et al. [16] also showed that dietary CGT supplementation enhanced broiler intestinal antioxidative capacity through promoting the expression of antioxidant genes, including *HO-1*, *CAT*, and *GPX1*, in a 42-d study. Yu et al. [33] investigated the effects of dietary TA supplementation on growth performance, diarrhea rate, and intestinal barrier integrity and function in weaned pigs, indicating that the addition of 1.0% TA decreased the ileal MDA concentration. Salman et al. [58] reported that TA treatment effectively up-regulated Nrf2/HO-1 pathway-related protein expression in brain tissue in mice. Moreover, a study in dairy cows also demonstrated that dietary chestnut tannin supplementation increased SOD activity in plasma and the liver [59]. Above all, adding 300 mg/kg CGT to the diet could relieve intestinal oxidative stress induced by the challenge of LPS through enhancing the antioxidant enzyme activity in broilers in this study.

## 5. Conclusions

In conclusion, the supplementation of 300 mg/kg CGT in broiler diets could effectively alleviate the LPS-induced adverse effects on the development and barrier function of the intestine. The positive effects of dietary CGT addition are partially a consequence of the reduction in cell apoptosis, enhancement of antioxidant capacity, and decrease in inflammatory responses in intestinal mucosa. Therefore, the supplementation of 300 mg/kg CGT may serve as a potential alternative to antibiotics for attenuating gut injury caused by LPS infection in broilers. Meanwhile, further study with a large-scale field trial is needed for evaluating the effectiveness of CGT in enhancing resistance to pathogenic bacteria in real-world production.

## Figures and Tables

**Table 1:** Ingredient composition and nutritional values of the basal diets (air-dried basis).

Items	Content
Ingredients, %	
Corn	55.90
Soybean meal, 44% CP	13.78
Wheat bran	11.98
Corn starch residue	7.99
Corn gluten meal	3.99
Extruded soybean	1.50
Limestone	1.70
Calcium monophosphate	1.10
L-Lysine HCL	1.00
DL-Methionine	0.20
L-Threonine	0.10
Sodium chloride	0.40
Choline	0.10
Phytase	0.10
Complex enzyme ^1^	0.02
Trace mineral premix ^2^	0.10
Vitamin premix ^3^	0.02
Antioxidant	0.02
Total	100
Calculated analysis, %	
Metabolizable energy, MJ/kg	12.33
Crude protein	19.47
Crude fat	3.45
Crude fiber	3.77
Calcium	0.94
Available phosphorus	0.35
Lysine	1.15
Methionine	0.50

^1^ Provided per kg of complete basal diet: amylase 4800 U, cellulase 200 U, glucanase 60 U, invertase 280 U, mannase 24 U, protease 480 U, and xylanase 480 U. ^2^ Provided per kilogram of complete basal diet: 10 mg of Cu as CuSO_4_, 100 mg of Fe as FeSO_4_, 1.1 mg of I as Ca (IO_3_)_2_, 65 mg of ZnSO_4_, 100 mg of Mn as MnSO_4_, and 0.3 mg of Se as Na_2_SeO_3_. ^3^ Provided per kilogram of complete basal diet: vitamin A 10,000 IU, vitamin D_3_ 3000 IU, vitamin E 30 IU, vitamin K_3_ 1.3 mg, vitamin B_1_ 2.2 mg, vitamin B_2_ 8 mg, vitamin B_3_ 8 mg, vitamin B_6_ 4 mg, vitamin B_12_ 0.025 mg, biotin 0.2 mg, niacin 40 mg, folic acid 1 mg, and D-calcium pantothenate 10 mg.

## Data Availability

All sequencing data are available in the NCBI Sequence Read Archive under accession PRJNA1040777 (Illumina sequences) at https://www.ncbi.nlm.nih.gov/bioproject/PRJNA1040777 (accessed on 15 November 2023).

## Supplementary Materials

The following supporting information can be downloaded at: https://www.mdpi.com/article/10.3390/ani14131915/s1, Table S1: Primer sequences used for quantitative real-time PCR; Table S2: Effects of Chinese gallotannins supplementation in the diet on microbial relative abundance at genus level (top 20) in cecal digesta of broiler chickens.
