# Supplementary material for: Effects of Chinese Gallotannins on Antioxidant Function, Intestinal Health, and Gut Flora in Broilers Challenged with Escherichia coli Lipopolysaccharide
Source: Animals (Basel). 2024 Jun 28;14(13):1915. doi: 10.3390/ani14131915 (PMC11240627; doi:10.3390/ani14131915)
Supplement: Supplementary file 1 [file animals-14-01915-s001.zip › animals-3046285-supplementary.pdf]

animals

MDPI

Supplementary Materials

Effects of Chinese Gallotannins on Antioxidant Function, Intestinal Health, and Gut Flora in Broilers Challenged with *Escherichia coli* Lipopolysaccharide

Yuemeng Fu [1], Peng Yuan [1], Nadia Everaert [2], Luke Comer [2], Shuzhen Jiang [1], Ning Jiao [1], Libo Huang [1], Xuejun Yuan [3], Weiren Yang [1] and Yang Li [1,2,*]

[1] Key Laboratory of Efficient Utilization of Non-Grain Feed Resources, Ministry of Agriculture and Rural Affairs, College of Animal Science and Technology, Shandong Agricultural University, Panhe Street 7, Tai'an 271017, China
[2] Division of Animal and Human Health Engineering, Department of Biosystems, KU Leuven, Kasteelpark Arenberg 30, 3001 Heverlee, Belgium
[3] College of Life Sciences, Shandong Agricultural University, Daizong Street 61, Tai'an 271018, China
* Correspondence: li_yang@sdau.edu.cn

Table S1. Primer sequences used for quantitative real-time PCR.

Genes	GenBank	Primer sequences, 5′-3′[1]	Size, bp
β-actin	NM_205518.1	F: ATTGTCCACCGCAAATGCTTC<br>R: AAATAAAGCCATGCCAATCTCGTC	113
ZO-1	XM_015278981.2	F: CCACCTCAGAATAAGCCAGCAAT<br>R: CGGTTGTAAGAAGGAGTGACTGTT	146
OCLN	NM_205128.1	F: ATCAACGACCGCCTCAATCAG<br>R: TCCTCTGCCACATCCTGGTATT	83
CLDN2	NM_001277622.1	F: ACATTGGTTCAAGCATCGTGAC<br>R: GCTGTAGATGTCGCACTGAGT	101
CLDN3	NM_204202.1	F: GCCAAGATCACCATCGTCTCC<br>R: ATCACCAGCGGGTTGTAGAAAT	116
GLUT2	NM_207178.2	F: TTTCGAGAGAGCCGGTGTTG<br>R: GCCTTCTCCACCAGGAAGAC	102
SGLT1	NM_001293240.1	F: CATCGTTATCCTGGCAGTCTCCTT<br>R: TCATCGGGTTTCTCCTCCTCATCA	138
y+LAT1	XM_418326.5	F: CTCTCTCTCATCATCTGGGC<br>R: TCATTCCTGGGTCTGTTGCT	472
FABP1	NM_204192.4	F: ACTGGCTCCAAAGTAATGACCAATG<br>R: TGTCTCCGTTGAGTTCGGTCAC	132
TLR4	NM_001030693.2	F: CATCTCTGGAGTTCCTGCTGAA<br>R: TGTATGGATGTGGCACCTTGA	145
MyD88	NM_001030962.5	F: CGGAGGATGGTGGTCGTCATT<br>R: TCGTTCTTCATGGTCTTGCACTTG	140
NF-κB	NM_001396038.1	F: CAGCCCATCTATGACAACCG<br>R: TCAGCCCAGAAACGAACCTC	152
Nrf2	MN416129.1	F: CACGCCTTGCTGTAGACTTCC<br>R: ATGAACTTGTGGCAGAGAGATGG	109
HO-1	NM_205344.2	F: GTCCCGAATGAATGCCCTTGA<br>R: ATGACCGTTCTCCTGGCTCTT	139
CAT	NM_001031215.2	F: GGAGGTAGAACAGATGGCGTATG<br>R: CGATGTCTATGCGTGTCAGGAT	114
SOD1	NM_205064.2	F: CGCAGGTGCTCACTTCAATCC	89

		R: CAGTCACATTGCCGAGGTCAC	
SOD2	NM_204211.2	F: GCTGTATCAGTTGGTGTTCAAGGA<br>R: GCAATGGAATGAGACCTGTTGTTC	130
GPX1	NM_001277853.3	F: CGGCTTCAAACCCAACTTCAC<br>R: CTCTCTCAGGAAGGCGAACAG	85

[1] F: forward primer; R: reverse primer. [2] ZO-1, zonula occludens-1; OCLN, occludin; CLDN2, claudin-2; CLDN3, claudin-3; GLUT2, glucose transporter type 2; SGLT1, sodium-glucose transporter 1; y+LAT1, y+L amino acid transporter-1; FABP1, fatty acid binding protein-1; TLR4, toll-like receptor 4; MyD88, myeloid differentiation primary response 88; NF-κB, nuclear factor-kappa B; Nrf2, nuclear factor erythroid 2-related factor 2; HO-1, heme-oxygenase 1; SOD1, superoxide dismutase 1; SOD2, superoxide dismutase 2; CAT, catalase; GPX1, glutathione peroxidase-1.

Table S2. Effects of Chinese gallotannins (CGT) supplementation in the diet on microbial relative abundance at genus level (top 20) in cecal digesta of broiler chickens.

Taxonomy, %	Treatments [1]			*P* value
	CON	LPS	LPS+CGT	
Alistipes	27.61 ± 6.00	22.58 ± 5.09	11.14 ± 3.75	0.092
Faecalibacterium	13.61 ± 4.35	6.98 ± 2.32	17.91 ± 6.65	0.295
Bacteroides	7.19 ± 2.89	8.22 ± 2.64	19.66 ± 9.79	0.304
Helicobacter	3.55 ± 2.75	5.80 ± 2.62	9.02 ± 3.43	0.441
Lactobacillus	0.27 ± 0.13	8.86 ± 8.45	0.13 ± 0.07	0.347
Ligilactobacillus	0.13 ± 0.03	4.67 ± 4.28	0.07 ± 0.04	0.344
Barnesiella	0.17 ± 0.05	1.95 ± 0.75	5.05 ± 3.14	0.207
Ruminococcus_torques_group	2.43 ± 0.75	1.31 ± 0.24	1.22 ± 041	0.216
CHKCI001	2.08 ± 1.74	0.26 ± 0.17	0.67 ± 0.30	0.441
Parabacteroides	2.48 ± 2.44	0.04 ± 0.01	0.18 ± 0.16	0.412
Colidextribacter	1.30 ± 0.35	1.09 ± 0.22	0.75 ± 0.07	0.297
Escherichia-Shigella	0.98 ± 0.46	2.79 ± 1.37	0.44 ± 0.13	0.149
Blautia	0.83 ± 0.36	0.78 ± 0.39	0.65 ± 0.19	0.920
UCG-005	0.47 ± 0.18	0.93 ± 0.36	1.01 ± 0.53	0.570
Subdoligranulum	0.33 ± 0.08	0.74 ± 0.52	0.25 ± 0.10	0.498
Phascolarctobacterium	0.18 ± 0.14	0.78 ± 0.48	0.13 ± 0.05	0.240
Enterococcus	0.16 ± 0.02	1.03 ± 0.71	0.10 ± 0.04	0.225
Fusobacterium	0.08 ± 0.03	2.76 ± 1.66	0.01 ± 0.01	0.101
UCG-008	0.02 ± 0.00	0.58 ± 0.38	0.67 ± 0.36	0.292
Sutterella	0.00 ± 0.00	0.00 ± 0.00	1.30 ± 1.28	0.381

[1] CON, broilers fed the basal diet and given intraperitoneal administration of saline solution; LPS, broilers fed the basal diet and given intraperitoneal administration of lipopolysaccharide (LPS); LPS+CGT, broilers fed the basal diet supplemented with 300 mg/kg CGT and given intraperitoneal administration of LPS. Values are mean ± standard error (n=6). Differences were considered statistically significant when $P < 0.05$.
